# Frequency distribution in intraoperative stimulation-evoked EMG responses during selective dorsal rhizotomy in children with cerebral palsy—part 1: clinical setting and neurophysiological procedure

**DOI:** 10.1007/s00381-020-04734-z

**Published:** 2020-06-23

**Authors:** Simone Wolter, Claudia Spies, John H. Martin, Matthias Schulz, Akosua Sarpong-Bengelsdorf, Joachim Unger, Ulrich-W. Thomale, Theodor Michael, James F. Murphy, Hannes Haberl

**Affiliations:** 1 Department of Anesthesiology and Operative Intensive Care Medicine (CCM, CVK), Charité – Universitätsmedizin Berlin, corporate member of Freie Universität Berlin, Humboldt-Universität zu Berlin, and Berlin Institute of Health, 13353 Berlin, Germany; 2 Department of Molecular, Cellular, and Basic Medical Sciences, Center for Discovery and Innovation, City University of New York School of Medicine, New York, NY USA; 3 Neuroscience Program, Graduate Center of the City University of New York, New York, NY USA; 4 Division of Pediatric Neurosurgery, Charité – Universitätsmedizin Berlin, corporate member of Freie Universität Berlin, Humboldt-Universität zu Berlin, and Berlin Institute of Health, 13353 Berlin, Germany; 5 Center for Chronically Sick Children (SPZ), Charité – Universitätsmedizin Berlin, corporate member of Freie Universität Berlin, Humboldt-Universität zu Berlin, and Berlin Institute of Health, 13353 Berlin, Germany; 6 Division of Pediatric Neurosurgery, Universitätsklinikum Bonn, 53127 Bonn, Germany; 7 Dahlem Research School, Freie Universität Berlin, 14195 Berlin, Germany

**Keywords:** Intraoperative neuromonitoring, SDR, Threshold intensity, Lumbosacral level differences, Rostro-caudal anatomical distribution, Stimulation-evoked EMG response

## Abstract

**Introduction:**

Selective dorsal rhizotomy (SDR) consists of microsurgical partial deafferentation of sensory nerve roots (L1–S2). It is primarily used today in decreasing spasticity in young cerebral palsy (CP) patients. Intraoperative monitoring (IOM) is an essential part of the surgical decision-making process, aimed at improving functional results. The role played by SDR-IOM is examined, while realizing that connections between complex EMG responses to nerve–root stimulation and a patient’s individual motor ability remain to be clarified.

**Methods:**

We conducted this retrospective study, analyzing EMG responses in 146 patients evoked by dorsal–root and rootlet stimulation, applying an objective response–classification system, and investigating the prevalence and distribution of the assessed grades. Part1 describes the clinical setting and SDR procedure, reintroduced in Germany by the senior author in 2007.

**Results:**

Stimulation-evoked EMG response patterns revealed significant differences along the segmental levels. More specifically, a comparison of grade 3+4 prevalence showed that higher-graded rootlets were more noticeable at lower nerve root levels (L5, S1), resulting in a typical rostro-caudal anatomical distribution.

**Conclusions:**

In view of its prophylactic potential, SDR should be carried out at an early stage in all CP patients suffering from severe spasticity. It is particularly effective when used as an integral part of a coordinated, comprehensive spasticity program in which a team of experts pool their information. The IOM findings pertaining to the anatomical grouping of grades could be of potential importance in adjusting the SDR-IOM intervention to suit the specific individual constellation, pending further validation.

**Trial registration:**

ClinicalTrials.gov ID: NCT03079362

## Introduction

The surgical technique known as selective posterior or dorsal rhizotomy (SDR) [1, 8, 9, 16], as it is currently termed, underwent a long, unsteady development, only gaining prominence in the 1980s [7, 17, 22, 34–36, 45, 47], in striving to provide long-lasting relief to infantile CP patients suffering from spastic muscle tonus, predominantly in the lower extremities [2, 4, 13, 19, 20, 24, 27, 30, 32]. Its revival at that time is commonly attributed to a number of technical innovations which, in fact, had been discussed by surgeons as early as the 1920s.

Today, SDR is gaining recognition as a safe and effective surgical procedure in treating CP patients. However, CP treatment varies between medical centers, as do procedures, depending on the expertise available. This especially applies when selecting patients for SDR.

Parents, intent on finding the best treatment for their child, frequently consult various independent experts, who point to numerous therapy options, leaving them confused in having to choose between contradictory and competing therapy options. Recognizing this, we have integrated SDR into a synergistic program which is agreed between a range of experts. Focusing on individual needs, a team of pediatric neurosurgeons, anesthesiologists, neuropediatricians, orthopedists, and physiotherapists customize therapy options in developing treatment plans. Subsequent to SDR, an in- or outpatient rehabilitation program helps facilitate the transition to everyday life.

Intraoperative monitoring (IOM), as used in SDR, has likewise undergone development over the years, with the latest knowledge [18, 25, 35, 41, 43] being applied in re-examining techniques [7, 26, 28, 36, 39, 47, 49] and bringing them up to date. We have come to realize how complex post-lesional changes can be [10, 21, 44, 48, 50] and have readjusted our expectations accordingly, with regard to the commonly anticipated responses.

Experience with the segmental distribution of EMG response patterns can be cited as one example. Going back to the earliest studies, it was assumed that individual nerve–root stimulation will only elicit responses in corresponding ipsilateral muscle groups. This assumption was weakened when overlapping innervation was repeatedly observed [36, 43], the fact that several adjacent muscle groups are also affected, which can even occur when direct stimulation is applied to a single ventral nerve root [43]. In our study, this concerned in particular the differentiation between grade 2 and grade 1 responses, which will be dealt with further on.

As mentioned, differences in neurosurgical and IOM procedures persist from center to center [47]. Thus, although the techniques we use are widely known [18, 25, 33, 37, 45, 49], we provide a brief summary in part 1, followed by a discussion of future prospects inspired by recent research findings. Part 2 reports unexpected findings obtained during IOM and their possible implications for clinical treatment.

## Methods

### SDR patient selection

The decision to carry out SDR is based on an optional MRI verification of the diagnosis [3] and a detailed clinical evaluation of the patient. Proceeding from the classic indication criteria applied slightly differently during two data acquisition stages in this study (from 2007 until about 2011 and from about 2012 until 2014), surgery is most strongly recommended for children who can walk with technical aids but without human assistance, corresponding to the Gross Motor Function Classification Scale [31] (GMFCS) and the Gross Motor Function Measure [40] (GMFM). Recently, children whose degree of impairment prevents them from walking have also seen improvements in their quality of life, but these patients are of no direct relevance to our study. Interviews with the parents are also designed to assess the child’s mental and emotional capacity to cope with the difficult perioperative period and the subsequent rehabilitation training process.

### Subjects

This retrospective study was approved by the responsible local ethics committee (approval number EA1/138/11) and the Official Data Protection Officer and was subsequently registered with the Clinical Trials Registry (ClinicalTrials.gov ID: NCT03079362). Informed consent was waived due to the retrospective nature of the approach and to the anonymity adhered to in the data processing used in this investigation.

Between January 2007 and December 2014, SDR was performed on a total of 148 children, 93 of whom underwent a physiotherapeutic evaluation (up to 2011, GMFM with a mean value of 78.5 ± 17% and thereafter of 74.3 ± 13%; GMFM dimensions: (D) standing and (E) walking, running, and jumping (GMFM-D&E) up to 2011 with a mean value of 59.5 ± 28%, and more recently of 45.5 ± 27%). GMFCS was applied in 99 children (predominantly GMFCS levels II and III starting in 2012 (up to 2011, levels I and IV were more common)).

In-house follow-up examinations had not yet become established practice when the first group of patients underwent SDR, and they were not carried out on out-patients who had to travel long distances.

SDR and IOM went hand in hand with all patients. In 146 of the 148 children treated, SDR was accompanied by thoroughly documented IOM, and the relevant data were encoded before being eligible for inclusion in the retrospective study analysis. Thus, our evaluations in part 1 and in part 2 were based on the results for 146 children with a mean age of 6.9 ± 3.1 years.

### Neurosurgical technique

In 2007, the senior author of this paper reintroduced in Europe the single-level approach developed by Park [33], in which a partial deafferentation of 50–70% of each sensory nerve root, from L1 to S2, is carried out close to the conus medullaris through a monosegmental laminectomy. A specific laminoplasty technique was added at that time [12].

Surgery is performed in prone position. After application of a single-shot of antibiotic prophylaxis, the lower part and the tip of the conus medullaris are exposed through an MRI-guided, single-level laminectomy. This access exposes the last root exits at the transition point, from the conus medullaris to the filum terminale, thereby providing an overview of the conus up to the dorsal root entry zones, at either L3 or L2, depending on the individual shape of the conus.

In the case of L1 exposure, the dural exit of L1 is identified. 50–60% of dorsal rootlets are sectioned without using IOM. Thereafter, the entire group of dorsal roots, beginning with L2, is gently lifted. A separating cotton is placed on the top of the more deeply seated motor nerve roots. The interspace between the sensory rootlets S1 and S2 is determined anatomically. A flexible plastic pad is pulled through this interspace underneath the dorsal roots S1 to L2, serving as a tray, which slightly lifts out the selected nerve roots and protects the remaining rootlets above L2 and below S2. Frequently, the conus is located at T12 or even higher. All corresponding sensory nerve roots above L1 then have to be shifted from the pad.

A partial deafferentation [33] of sensory nerve roots L1 to S2 is performed, proceeding from rostral to caudal. In the process, each nerve root is successively subdivided into at least 4 component rootlets and each rootlet is tested individually through IOM (Data acquisition: stages I–III). At the outset of our SDR intervention program, only nerve roots L1 to S1 were treated, nerve root S2 being included [22] later on. Deafferentation was not always initiated on the same side, but on the right side with 89 patients, on the left side with 57. The nerve roots are identified anatomically. Applying electric current stimulation and mechanical tapping may help to confirm the level and to prevent the accidental involvement of a motor nerve root.

Based on the assessments obtained, about 50 to 70% [27, 33] of the rootlets of each nerve root are transected, preference being given to rootlets which showed the higher-grade responses described below.

Finally, S1, S2 and S3 are assessed with regard to pudendal dorsal-root action potential [18] (pDRAP), and the lower-rated 50% of S2 are transected (see Data acquisition: stage III). After dura closure, an epidural catheter is put in place for pain management. The removed lamina is refixed and a compression suture fuses the bisected neighboring spinous processes [12].

### Anesthetic procedure

In adapting the anesthetic procedure to IOM requirements, in particular those pertaining to triggered EMG, muscle relaxants were administered once only, in order to facilitate tracheal intubation. During the operation, following Riegle’s recommendation [38], a balanced form of anesthesia was used on all patients, in which sevoflurane (1.0–1.1 vol% in oxygen:air mixture) was combined with continuous remifentanil (0.3–0.5 μg kg^−1^ min^−1^), a technique which has been used successfully by another operating team during SDR [23].

### Intraoperative neuromonitoring

The decision as to which rootlets of a nerve root are to be transected is determined by IOM, which accompanies each microsurgical intervention [33]. Responses are elicited through intraoperative electrical nerve-root stimulation [33, 37] at the previously determined threshold intensity. We deliver a 1-second, high-frequency stimulation (current-controlled) [28] at this threshold intensity, examining and recording threshold values and EMG responses elicited in the lower-limb muscle groups through this rootlet stimulation. The observed EMG patterns are then inspected [45] and grouped into grades [33, 37]. In turn, each stimulated rootlet is graded on the basis of the threshold EMG response, according to a five-level grading scheme devised by Phillips and Park [37] and slightly adapted in Park and Johnston [33]. Grades 0 to 4+ are described below. The higher the grade in this objective response-classification scale, the more intense and/or extensive the EMG responses are [8, 34, 45, 49]. The occurrence, in absolute numbers and relative frequency, of the five different grades and their location served as starting point in the subsequent statistical evaluations presented in part 1 and part 2.

All rhizotomies were monitored [33] with the help of special IOM equipment (Cascade Elite, Cadwell Inc., Kennewick, USA/Eclipse, Axon Inc., Hauppauge, USA). The EMG responses, which were triggered by the 1-second 50-Hz train stimulation applied at dorsal rootlets adjacent to the conus medullaris and which subsequently appeared in relevant muscle groups [36, 43], were recorded by pairs of subdermal needle electrodes, inserted bilaterally (predominant selection: AL (adductor longus), VA (vastus medialis), TA (tibialis anterior), BF (biceps femoris), PL (peroneus longus), MG (gastrocnemius), Gra (gracilis), Sol (soleus), AH (abductor halluces), EAS (external anal sphincter). In the frequently used application, the active needle was inserted intramuscularly, the reference electrode subdermally above it). The frequency range of the high- and low-pass filters was set between 10 Hz and 10 kHz, while the free-running multichannel EMG (200–300-ms/div sweep length; 50–200-μV/div gain) was observed throughout.

### Data acquisition

The following section describes the monitoring procedure for each side of the body and for the nerve roots L2 to S1/S2 and their rootlets, based on widely known SDR neuromonitoring techniques [18, 25, 33, 37, 45]:

I. Determining required threshold intensity of stimulation current: The focus was on the distribution of EMG patterns in corresponding muscle groups, following orthodromic single stimulation, applying a monophasic [28] 0.1-ms rectangular wave pulse [36] at 0.7–0.8 Hz. Two monopolar (inter-electrode distance, about 10 mm), hand-held, hooked probes (Dr. Langer Medical GmbH, Waldkirch, Germany) with firm connecting plug were used to stimulate dorsal nerve roots and rootlets adjacent to the conus medullaris. The minimal threshold intensity, defined as the current needed to elicit a just noticeable response potential (CMAP, compound muscle action potential), was determined by increasing the current intensity incrementally (from 0.1 mA to a seldom reached maximum of 10 mA) until a response potential was evoked (recorded with the following display settings: 4–5-ms/div sweep length; 100–200-μV/div gain).
II. Train stimulation and grading of 50-Hz response: Using the previously identified threshold intensity (stage I), a 1-second, 50-Hz train stimulation was delivered at the same spot in the rootlet, following which the elicited 50-Hz responses were classified [33, 37] according to the data analysis criteria described in the next section. In a few cases, stimulation was repeated in order to verify assessments. The stimulation intensity was deliberately limited to a maximum of 7 mA, especially in the case of grade 4. The evoked 50-Hz EMG responses were recorded from both sides of the body (display settings: 200–300-ms/div sweep length; 50–200-μV/div gain).
III. Determining distribution of pDRAP activity: After nerve root S2 was included in SDR, the pudendal dorsal-root action potential [18] of two S2 rootlets was compared, in order to determine which of the two showed less pDRAP activity. It was likewise important to test, through pudendal stimulation (repeated at a rate of 13.3 Hz; 0.2-ms pulse duration), whether nerve roots S1 and S3 also transmitted activity. The frequency range of the high- and low-pass filters was set between 1.5 Hz and 2.1 kHz, and the pDRAP activity (100 sweeps) was recorded (2–5-ms/div sweep length; 10-μV/div gain).

### Data analysis

Here we describe the individual steps which were followed in assessing the 50-Hz responses (Data acquisition: stage II) and the subsequent classification of individual rootlets at the time of SDR intervention.

Grade classification is based on the above-mentioned scale [33, 37], which is among the generally agreed guidelines [25] governing IOM and is widely used in SDR surgery. This objective grading [49] was carried out for each rootlet of the root in question. The classification of rootlets into the five specific grades depended on the nature and distribution of 50-Hz responses, ranging from grade 0 (no abnormalities) to grade 4+ (sustained contralateral abnormal patterns; see Data processing).

In addition, during the SDR intervention, thresholds and specific EMG patterns [45] were taken into account (i.e., waxing or waning). Finally, attention was paid to special features of IOM patterns (CMAP) which may be of interest but play little or no role in the decision-making process at this time.

### Data processing

In the data preparation, selected data pertaining to each rootlet and nerve root, taken separately for each body side and compiled in the IOM log, were incorporated anonymously into a local database as variables in statistical analysis: (1) number of rootlets tested per nerve root; (2) number of rootlets classified as grade 0, grade 1, grade 2, grade 3, or grade 4 respectively; (3) segmental distribution of assessed grades 1 to 4+ (sustained responses).

At a later stage, a merger was carried out. As grade 4 occurred relatively rarely, it was grouped together with the next lower grade (grade 3) as *grades 3+4* for statistical purposes, thus conjoining the two degrees of greatest severity. Grade 2 was often difficult to assess because of radicular overlap [36, 43] when examining whether nerve root stimulation caused innervation of adjacent muscle groups. For this reason, grade 1 was combined with grade 2 as *grades 1+2*. In accordance with the criteria defined for each grade [33, 37, 49], and referring to the respective nerve root stimulated, the following characteristic description of 50-Hz activity was arrived at, after results for the two groups were merged:

- Grade 0: single discharge, or absence of abnormalities and responsiveness to 50-Hz stimulation
- Grades 1+2: sustained discharge, appearing ipsilaterally at the same innervated (grade 1) and/or adjacent (grade 2) myotome
- Grades 3+4: sustained, ipsilateral, widely spread discharges (grade 3), often at multiple levels, and/or contralateral (grade 4) EMG response

As the S2 nerve root was not initially included in the surgical intervention, only nerve roots L2–S1 were considered in the statistical analysis. pDRAP values were not included in the evaluation.

### Statistical analysis

The investigation was limited to intraoperatively evaluated grade results taken from the IOM logs. A descriptive assessment of the entire sample was undertaken, first using graphics showing median, mean values and cross-tabulations, followed by chi-square (***χ***^2^) tests. Mean values plus standard deviation (mean ± SD) appear in the text and tables. As is common practice in descriptive overviews, mean values were chosen for the bar chart here in part 1.

The relative grade values, given as percentages, were assessed with regard to the homogeneity of distribution over the five nerve-root levels involved (part 1) and side independency (part 2). A non-parametric procedure with repeated measures was carried out, as devised by Brunner and Munzel [5], using 3- and 2-factorial variance analysis (ANOVA). The date of SDR initiation and the start-up side (i.e., whether surgery began on the right or left) were considered in conjunction with the main factors of level and side. Initial findings from a smaller sample, intended as a hypothesis-generating interim evaluation, were considered before assessments were made.

The SAS 9.4 application (SAS Institute Inc., Cary, USA) and IBM SPSS Statistics for Windows version 21.0 (IBM Corp., Armonk, USA) were used throughout. Significance level *α* = 0.05 was chosen as the basis for all the statistical tests. Our approach in interpreting the results was explorative, hence there was no need for an alpha adjustment.

## Results

**Fig. 1:**
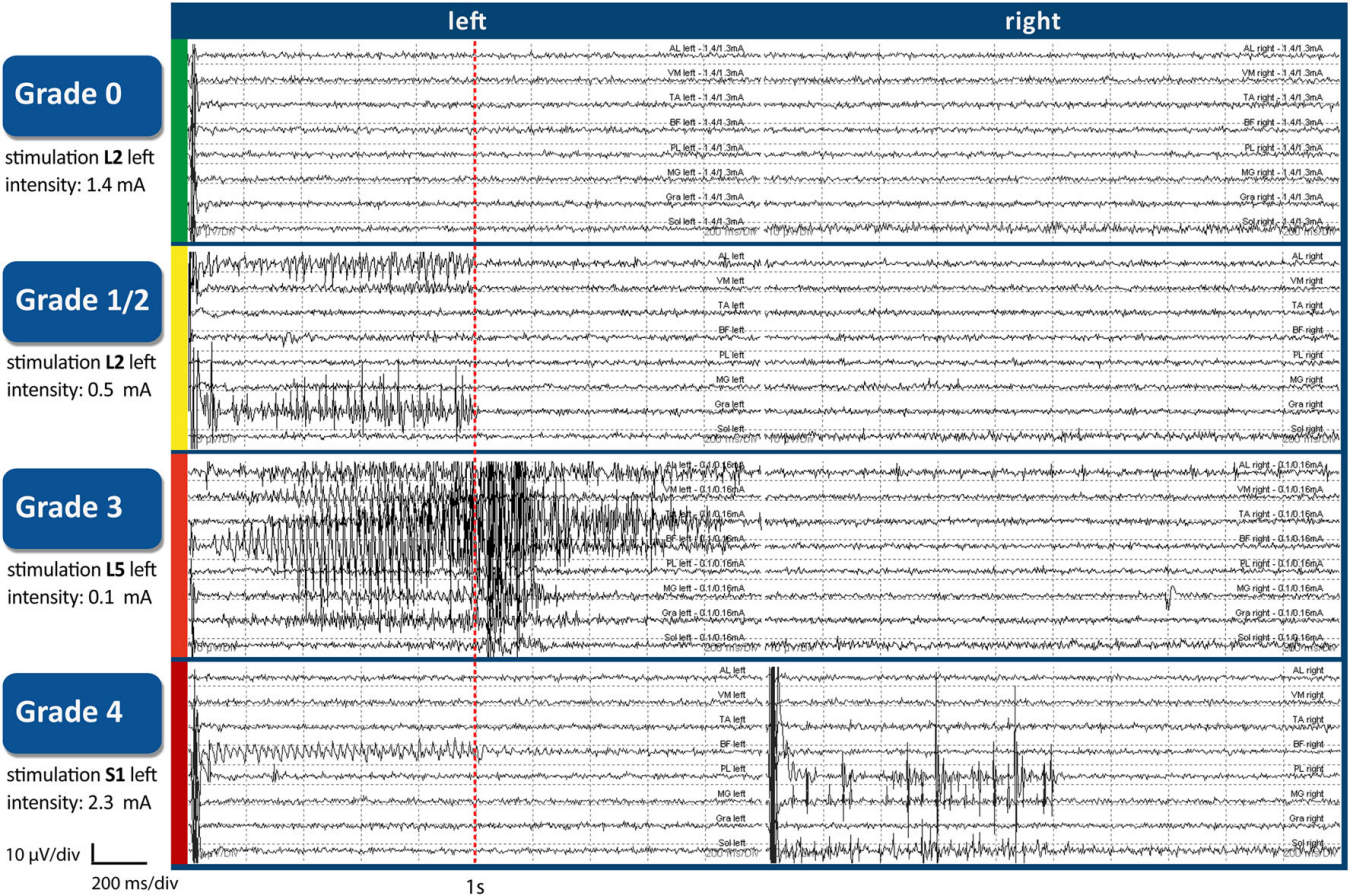
EMG patterns showing how the assessment of responses for specific grades was undertaken, using the Phillips and Park’s scale (slightly adapted in Park and Johnston 2006), following 50-Hz train stimulation of a rootlet after its threshold intensity was determined. The color coding in the vertical axis of the graph illustrating EMG patterns corresponds with that used in marking the bar chart (Fig. 2 in this part) and the three segments in the pie charts (Fig. 2 in part 2): grade 0 (green) = absence of abnormalities and responsiveness to 50-Hz stimulation; grade 1/2 (yellow) = sustained discharges, appearing ipsilaterally at the same innervated (grade 1) and/or adjacent (grade 2) myotome; grade 3 (red) = sustained, widely spread discharges; and grade 4 (red) = with contralateral spread. A detailed description of the individual grades and the muscle groups selected for this EMG recording (topdown: AL, VA, TA, BF, PL, MG, Gra, Sol) can be found in this part 1 in the section Intraoperative neuromonitoring

**Fig. 2:**
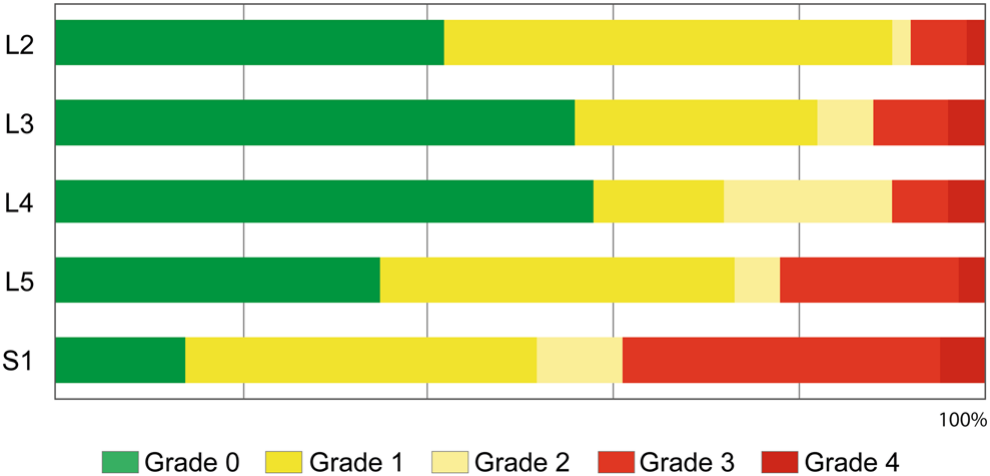
The bar chart shows the proportional mean frequency of the grades (0–4) within a certain nerve root (rostro-caudally aligned), averaged over the complete sample. Thus, differences involving the rostro-caudal distribution of grades are illustrated. The color code is explained at the bottom and corresponds to the grading categories (Fig. 1). A comparison of grade 3 and grade 4 prevalence (marked in two shades of red) shows that higher-graded rootlets were more noticeable at lower nerve root levels (L5, S1). The rootlets which showed inconspicuous grade 0 response, marked in green, were more frequent in L3 and L4. The color marking is almost identical for grade 1 and grade 2 (marked in two shades of yellow) since radicular overlap often makes it difficult to determine whether it is the corresponding muscle groups that are responding to stimulation or an adjacent group [36, 43].

In our complete sample of 146 children, the EMG patterns elicited through stimulation of dorsal nerve roots in the lower-limb-muscle groups were visually inspected and graded (step II in description of the IOM procedure). The elicitation of higher-grade responses to 50-Hz stimulation was the most important factor in deciding which rootlets to transect. Lower-graded rootlets might also be transected, the degree of dissection being fixed at between 50 and 70%. Figure 1 (the graph illustrating EMG patterns) indicates the general context, showing the different grades. The higher the grade, the more one can assume increased responsiveness to 50-Hz train stimulation [8, 34, 45, 49]. The bar chart in Fig. 2 shows the proportional mean frequency of the grades per nerve-root level over the complete sample. In individual cases, intraoperatively assessed EMG grade distribution can deviate from the mean. Only occasionally do more than one or two grades occur for rootlets within a single nerve root. When all the component rootlets within a nerve root were similar in grade, which was not infrequent, thresholds and specific EMG patterns [45] were taken into account. If grading yields inconclusive assessments at nerve root S2, the rootlet showing a lower percentage of pDRAP activity [18] is transected.

A total of 7018 rootlets (left 3503/ right 3515) were classified according to EMG responses during the SDR intervention. In terms of absolute numbers, the grades assessed per level were very differently distributed (Table 1). Grade 0 signifies the absence of abnormalities and responsiveness to 50-Hz stimulation, and 2835 rootlets (left 1424/right 1411) received this classification. In 4183 rootlets (left 2079/right 2104), the evoked responses deviated from grade 0 and showed sustained response patterns (grades 1 to 4; partially merged to form grades 1+2 and grades 3+4). Thus, there were 2856 rootlets (left 1325/right 1531) showing grade 1+2 responses and 1327 rootlets (left 754/right 573) in grades 3+4. In particular, those rootlets showing sustained and widely spread responses to stimulation were not homogeneously distributed. With regard to grade 3+4 occurrence during the interim evaluation, we tested whether the significant body-side effect [51] might have been caused by repeated initiation of deafferentation on the same side, but results from our initial small sample showed no significance or interactions (ANOVA, *p* = 0.12; to date, unpublished result).

**Table 1:** Absolute frequency and relative mean frequency (%) of grades recorded for rootlets in each nerve-root level tested

Number of rootlets assessed						
Level	Total	Grade 0 (%)	Grade 1 (%)	Grade 2 (%)	Grade 3 (%)	Grade 4 (%)
L2	1225	514 (41.9 ± 34)	582 (47.5 ± 34)	23 (2.0 ± 6.7)	78 (6.2 ± 14)	28 (2.5 ± 10)
L3	1252	703 (56.4 ± 33)	322 (25.7 ± 26)	76 (5.9 ± 14)	98 (7.9 ± 15)	53 (4.2 ± 13)
L4	1367	813 (57.4 ± 32)	188 (13.9 ± 23)	241 (18.3 ± 23)	77 (6.3 ± 14)	48 (4.0 ± 12)
L5	1554	565 (35.4 ± 31)	589 (38.4 ± 27)	66 (4.2 ± 11)	294 (19.4 ± 25)	40 (2.6 ± 9)
S1	1620	240 (13.9 ± 22)	648 (38.4 ± 33)	121 (8.5 ± 20)	544 (34.5 ± 34)	67 (4.7 ± 12)
*Σ*	7018	2835	2329	527	1091	236

Additional results in part 2 reaffirmed that the grades were not evenly distributed. In the main investigation in that part, highly significant (*p* < 0.001) differences were consistently observable across the various lumbosacral levels (L2–S1).

## Discussion

Detailed insight into the patient’s initial spinal-neurofunctional state prior to deafferentation is made possible through IOM, which is an integral part of the single-level intervention we perform. IOM was used in assessing EMG responses and classifying them intraoperatively into grades in order to decide which rootlets of a nerve root to transect. The information on exacerbated stimulation-evoked EMG response patterns recorded from the lower-limb muscle groups is thus crucial for two reasons. Firstly, it affects the decision as to whether a particular rootlet is to be transected. Secondly, it sheds light on the reflex state at the time of the SDR, thus providing information on the reorganization process.

In our investigations, we focused particularly on the anatomical distribution of the nearly 60% of rootlets showing sustained responses, particularly the approximately 32% with grades 3+4 (Table 1, Fig. 2), marked by sustained and widely spread EMG patterns, suggesting increased excitation. Proceeding from there, a partial section per nerve root was performed, preference being given to these higher-graded rootlets (highest degrees: grades 3+4, the red marked EMG patterns in Fig. 1). Of equal significance was the segmental distribution of the more than 40% of rootlets which showed inconspicuous grade 0 response (Fig. 2) in our entire sample, consisting of 146 CP children.

At the earliest stages of this study, the assumption prevailed that there was no specific pattern in the distribution of EMG response grades across lumbosacral levels from L2 to S1, justifying random dissection approaches. However, our initial findings [51] already revealed definite lateral and rostro-caudal distribution patterns. A comparison of grade 3+4 prevalence confirmed that there were noticeable differences over these various levels.

### Distribution of exacerbated stimulation-evoked EMG responses along the rostro-caudal levels

Highly significant grade differences were observed in individual segmental levels. To our knowledge, no comparable results for similarly large patient numbers have been published to date, nor has an anatomical grouping of grades (rostro-caudally aligned) following 50-Hz dorsal-root and rootlet stimulation been attempted elsewhere. At the same time, there remains room for refinement, as several researchers have suggested [6, 46].

A number of past studies investigating segmental variations between upper-lumbar nerve root L2 and sacral nerve roots S1/S2 have focused primarily on the proportion of rootlets to be transected during rhizotomies, without looking closely at each individual lumbosacral level or considering the factors of gender, body side and physiotherapeutic evaluations (GMFCS levels), as we do in part 2. Those articles which provide details on individual lumbosacral nerve roots and the portion of rootlets to be transected, based on the abnormal responses observed, might be compared with those of ours in which such sustained grade 3+4 patterns were noted (see the red marked EMG patterns in Fig. 1). Valuable information has been provided by Staudt et al. [45], Morota [30], Hays et al. [17], and Fukuhara et al. [11] in this context. For Fukuhara’s research team, palpating responses (clonic and bilateral) were the decisive factor in determining what rootlets to transect. They [11] reported that larger proportions of rootlets were sectioned at L5 and S1 (lower nerve- root levels) and smaller ones at L4 and L3 (next higher segments), which is in keeping with differences we have detected with regard to the occurrence of grades 3+4 at the different lumbosacral levels (Fig. 2), although no direct comparison can be made.

In the data presented by McLaughlin and Hays et al. [17] indicating the proportion of abnormally responding rootlets partly averaged over several nerve-root levels, significantly more rootlets with abnormal responses were also reported in the lower segments. This led them to suspect that the sectioning process itself—the fact that it was initiated caudally and advanced rostrally—might have caused a desensitizing of the upper-lumbar nerve roots. However, although we proceeded from the opposite end, our results were similar to theirs. The same applied to the side of the body. No relation was found between where the transection began and the prevalence of higher-grade responses.

It seems unlikely that the sequence in which sectioning proceeds influences subsequent stimulation responses. The point in time in which the primary insult occurred and the extent of damage most likely determine the degree of post-lesional reorganization [10] in the corticospinal tracts, a process which can last a lifetime.

Discussing the three impairment mechanisms in patients with chronic paresis (paresis, soft tissue contracture, and muscle overactivity), Gracies [15] emphasizes that none of them are distributed symmetrically and that when the leg muscles are tensed, hamstrings, which are innervated by lower nerve-root levels, show proportionately more activation than quadriceps, which are innervated by upper-lumbar nerve roots. These assessments might possibly correspond with our findings regarding the varying distribution of grades between upper-lumbar (L2/L3) and lower nerve-root levels (L5/S1), observed intraoperatively. Parallels of this kind between observed clinical results [15] and the elevated responses recorded through IOM, evoked by L5/S1-rootlet stimulation, represent an important accord.

Despite these parallels, our results raise questions about so-called tailored rhizotomy, in which only certain reference muscles and/or nerve roots are treated. Our SDR findings strongly suggest—at least with regard to patients with preoperative GMFM results between 65 and 85% [13] benefitting the most—that cutting the same proportion of rootlets across all nerve-root levels [27, 32] leads to comparable functional outcomes for each single muscle in the lower limbs. In view of this, we decided against confining SDR to grade 3 and grade 4 rootlets, while continuing to give preference to them when cutting.

At the same time, more consideration might be given to individual needs, as demonstrated in a new approach [29] in which preoperative clinical evaluation results (GMFM, GMFCS level) are given careful attention, resulting in significant positive changes in the GMFCS level I group and producing improved post-SDR outcomes.

Generally speaking, in refining the dissection procedure, numerous multilevel effects should be considered, including propriospinal reflexes which affect all spinal-cord segmental levels bilaterally [44]. Although the severing of dorsal nerve fibers is a localized intervention, confined to individual segments, it no doubt has wide-ranging, suprasegmental effects, improving corticospinal functioning, normalizing reflexes in the lumbosacral spinal cord, and reducing excitability in intersegmental circuits.

SDR has been reported to have benefits that go beyond lumbosacral function, including improvements in the upper extremities [14], as well as in speech and in cognitive functioning [42], particularly in more severely handicapped CP children.

**Fig. 3:**
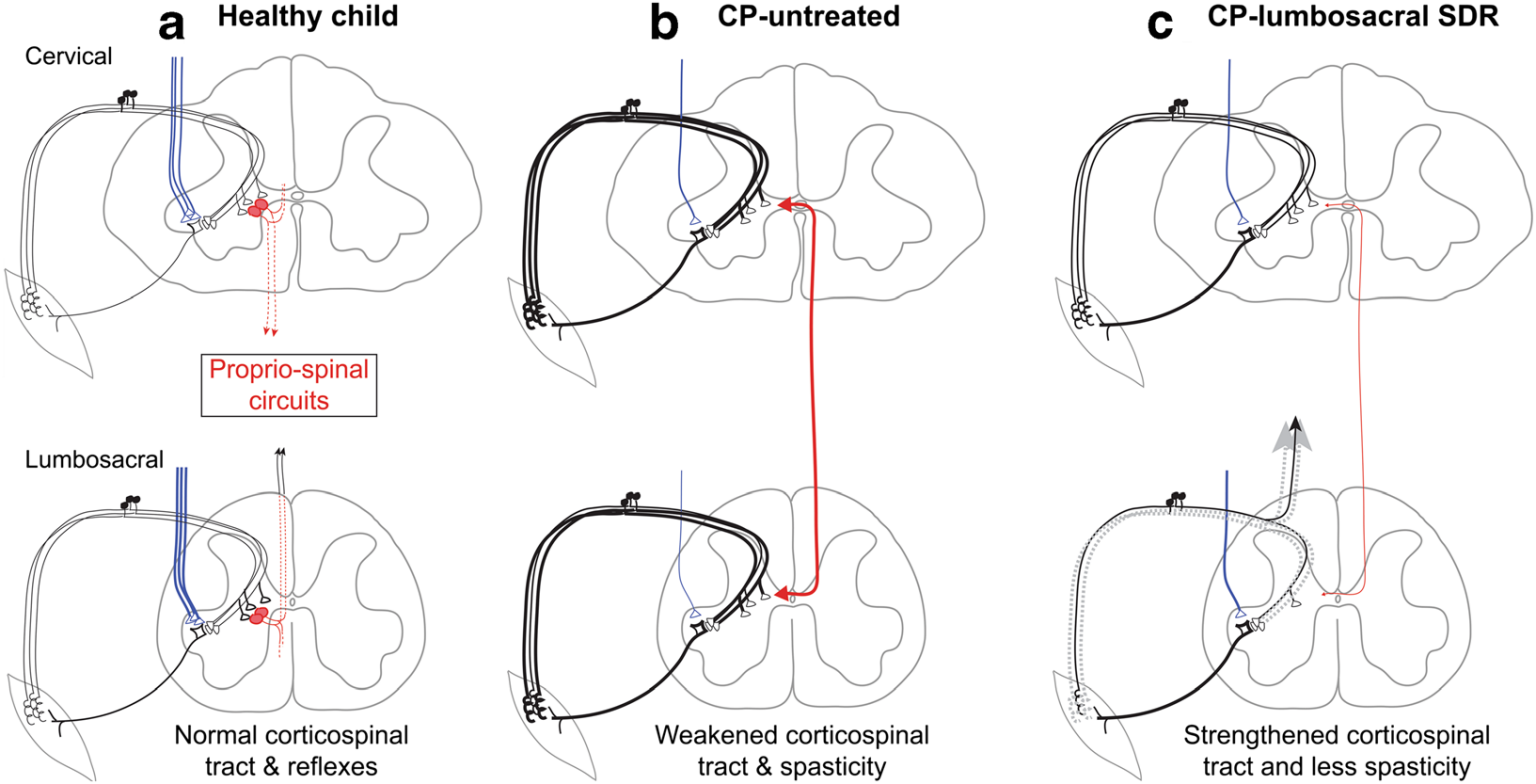
Model illustrating changes observed in motor function following SDR. Each panel contains the drawing of a cervical and lumbosacral spinal cord segment. The sensorimotor reflex circuit is shown, with several 1A afferent fibers converging on a single motoneuron. The intersegmental propriospinal circuit, illustrated schematically, interconnects spinal circuits in the cervical and lumbar levels. The descending corticospinal tract projection is in blue. The thickness of a line represents the physiological state of the system. **a** Healthy child. The reflex, propriospinal, and corticospinal circuits are in balance. **b** CP—untreated. Developmental brain injury results in partial loss of the corticospinal projection. This loss, together with other factors, leads to hyper-excitability in segmental reflex circuits (thick black lines) as well as to enhanced excitability of intersegmental circuits (thick red line). **c** CP—lumbosacral SDR. SDR involves sectioning sets of rootlets that, on stimulation, show abnormal muscle responses (dotted gray lines correspond to the sectioned rootlets). The result is reduced hyperreflexia and a compensatory improvement in corticospinal system motor functions. It is proposed that the combined improvement in corticospinal functions together with the normalization of reflexes in the lumbosacral spinal cord leads to reduced excitability of intersegmental circuits and, in turn, improvements in the cervical spinal cord

Our model (Fig. 3) shows how lumbosacral SDR can improve upper extremity and cervical spinal motor functions [50]. The damaged motor system in a child with CP is characterized by fewer descending corticospinal tract fibers in the spinal-cord white matter as a result of the brain injury (single fiber in tract, Fig. 3b, compared with many in Fig. 3a). This loss causes a reactive increase in the density of dorsal nerve fibers, specifically those from muscles that are most directly involved in reflex function [21, 48, 50]. This is shown (compare Fig. 3b with Fig. 3a) as thicker dorsal nerve fibers in both the cervical and lumbosacral spinal levels. Spinal reflex circuits become hyperactive/hyperreflexive following a corticospinal tract lesion [48]. We postulate that propriospinal interconnections reflect this increased activity (Fig. 3b, thick line) and that lumbosacral SDR of hyperactive dorsal nerve fibers (Fig. 3c, lumbosacral) can thus ameliorate the hyperreflexia, thereby helping to reduce intersegmental hyperactivity and, correspondingly, to improve cervical cord motor function. Ongoing improvements in limb motor function might also improve cognitive functions.

All of these observations relating to children with CP will hopefully contribute to a better understanding of the complex multilevel spinal nerve-root repair mechanisms that go into operation in the wake of a cerebral insult and help us fine tune the SDR-IOM procedure to more closely fit individual needs.

### Limitations

See part 2 with regard to the entire retrospective cohort study.

## Conclusions

Clinical results encourage us to consider SDR as a first-line prophylactic treatment for all CP patients. IOM represents a link between clinical practice and basic research. The interpretation of IOM features—informed by research results in the spinal-repair mechanisms after cerebral insults—may improve the surgical decision-making process.

Thus, specific IOM patterns and muscle activation evoked by rootlet stimulation should be carefully noted during the SDR-IOM intervention, as comprehensively monitoring the physiology and pathophysiology of the rootlets might provide useful information on cardinal CP signs, in particular hyperreflexia and the co-activism of antagonist muscles.

## Abbreviations

ANCOVA: analysis of covariance
ANOVA: analysis of variance
CMAP: compound muscle action potential
CP: cerebral palsy
EMG: electromyogram
GMFCS: Gross Motor Function Classification System
GMFM: Gross Motor Function Measure
GMFM-D&E: Gross Motor Function Measure, dimensions: standing [D] and walking, running, jumping [E]
IOM: intraoperative neuromonitoring
L1-L5: lumbar nerve-root levels
pDRAP: pudendal dorsal root action potential
*p* value: probability value
S1-S3: sacral nerve-root levels
SDR: selective dorsal rhizotomy
***χ***^2^ test: chi-square tests

## References

[1] Abott R, Forem SL, Johann M (1989). Selective posterior rhizotomy for the treatment of spasticity: a review. Childs Nerv Syst.

[2] Ailon T, Beauchamp R, Miller S, Mortenson P, Kerr JM, Hengel AR, Steinbok P (2015). Long-term outcome after selective dorsal rhizotomy in children with spastic cerebral palsy. Childs Nerv Syst.

[3] Ashwal S, Russman BS, Blasco PA, Miller G, Sandler A, Shevell M, Stevenson R (2004). Practice parameter: diagnostic assessment of the child with cerebral palsy: report of the quality standards subcommittee of the American Academy of Neurology and the Practice Committee of the Child Neurology Society. Neurology.

[4] Bolster EA, van Schie PE, Becher JG, van Ouwerkerk W, RLM S, Vermeulen RJ (2013). Long-term effect of selective dorsal rhizotomy on gross motor function in ambulant children with spastic bilateral cerebral palsy, compared with reference centiles. Dev Med Child Neurol.

[5] Brunner E, Munzel U (2002). Nicht-parametrische Datenanalyse.

[6] Cohen AR, Webster HC (1991). How selective is selective posterior rhizotomy?. Surg Neurol.

[7] Farmer JP, Sabbah AJ (2007). Selective dorsal rhizotomies in the treatment of spasticity related to cerebral palsy. Childs Nerv Syst.

[8] Fasano VA, Broggi G, Zeme S (1988). Intraoperative electrical stimulation for functional posterior rhizotomy. Scand J Rehabil Med Suppl.

[9] Förster O (1908). Über eine neue operative Methode der Behandlung spastischer Laehmungen mittels Resektion hinterer Rueckenmarkswurzeln. Z Orthop Chir.

[10] Friel KM, Williams PT, Serradj N, Chakrabarty S, Martin JH (2014). Activity-based therapies for repair of the corticospinal system injured during development. Front Neurol.

[11] Fukuhara T, Najm IM, Levin KH, Luciano MG, Brant CL (2000). Nerve rootlets to be sectioned for spasticity resolution in selective dorsal rhizotomy. Surg Neurol.

[12] Funk J, Haberl H (2016). Monosegmental laminoplasty for selective dorsal rhizotomy - operative technique and influence on the development of scoliosis in ambulatory children with cerebral palsy. Childs Nerv Syst.

[13] Funk JF, Panthen A, Bakir MS, Gruschke F, Sarpong A, Wagner C, Lebek S, Haberl EJ (2015). Predictors for the benefit of selective dorsal rhizotomy. Res Dev Disabil.

[14] Gigante P, McDowell MM, Bruce SS, Chirelstein G, Chiriboga CA, Dutkowsky J, Fontana E, Hyman J, Kim H, Morgan D, Pearson TS, Roye BD, Roye DP, Ryan P, Vitale M, Anderson RC (2013). Reduction in upper-extremity tone after lumbar selective dorsal rhizotomy in children with cerebral palsy. J Neurosurg Pediatr.

[15] Gracies JM (2005). Pathophysiology of spastic paresis. I: paresis and soft tissue changes & II: emergence of muscle overactivity. Muscle Nerve.

[16] Gros C, Ouaknine G, Vlahovitch B, Frerebeau P (1967). La radicotomie sélective postérieure dans le traitement neuro-chirurgical de l’hypertonie pyramidale. Neurochirurgie.

[17] Hays RM, McLaughlin JF, Stephens K, Price R (1998). Electrophysiological monitoring during selective dorsal rhizotomy, and spasticity and GMFM performance. Dev Med Child Neurol.

[18] Huang JC, Deletis V, Vodusek DB, Abbott R (1997). Preservation of pudendal afferents in sacral rhizotomies. Neurosurgery.

[19] Hurvitz EA, Marciniak CM, Daunter AK, Haapala HJ, Stibb SM, McCormick SF, Muraszko KM, Gaebler-Spira D (2013). Functional outcomes of childhood dorsal rhizotomy in adults and adolescents with cerebral palsy. J Neurosurg Pediatr.

[20] Ingale H, Ughratdar I, Muquit S, Moussa AA, Vloeberghs MH (2016). Selective dorsal rhizotomy as an alternative to intrathecal baclofen pump replacement in GMFCS grades 5 and 5 children. Childs Nerv Syst.

[21] Jiang YQ, Zaaimi B, Martin FH (2016). Competition with primary afferents drives remodeling of corticospinal axons in mature spinal motor circuits. J Neurosci.

[22] Kim DS, Choi JU, Yang KH, Park CI, Park ES (2002). Selective posterior rhizotomy for lower extremity spasticity: how much and which of the posterior rootlets should be cut?. Surg Neurol.

[23] Konya D, Gercek A, Dagcinar A, Baykan N, Ozek MM (2009). Prevention of brisk hyperactive response during selective dorsal rhizotomy in children with spasticity: isoflurane versus sevoflurane maintenance anesthesia. J Clin Neurosci.

[24] Langerak NG, Lamberts RP, Fieggen AG, Peter JC, Peacock WJ, Vaughan CL (2009). Functional status of patients with cerebral palsy according to the International Classification of Functioning Disability and Health model: a 20 year follow-up study after selective dorsal rhizotomy. Arch Phys Med Rehabil.

[25] Leppanen RE (2005). Intraoperative monitoring of segmental spinal nerve root function with free-run and electrically-triggered electromyography and spinal cord function with reflexes and F-responses. J Clin Monit Comput.

[26] Logigian EL, Soriano SG, Herrmann DN, Madsen JR (2001). Gentle dorsal root retraction and dissection can cause areflexia: implications for intraoperative monitoring during selective partial dorsal rhizotomy. Muscle Nerve.

[27] McLaughlin JF, Bjornson KF, Temkin N, Steinbok P, Wright V, Reiner A, Roberts TS, Drake J, O’Donnell M, Rosenbaum P, Barber J, Ferrel A (2002). Selective dorsal rhizotomy: meta-analysis of three randomized controlled trials. Dev Med Child Neurol.

[28] Merrill DR, Bikson M, Jefferys JGR (2005). Electrical stimulation of excitable tissue: design of efficacious and safe protocols. J Neurosci Methods.

[29] Morota N (2019). Clinically practical formula for preoperatively estimating the cutting rate of the spinal nerve root in a functional posterior rhizotomy. Childs Nerv Syst.

[30] Morota N (2007). Functional posterior rhizotomy: the Tokyo experience. Childs Nerv Syst.

[31] Palisano R, Rosenbaum P, Walter S, Russell D, Wood E, Galuppi B (1997). Development and validation of a gross motor function classification system for children with cerebral palsy. Dev Med Child Neurol.

[32] Park TS, Dobbs MB, Cho J (2018). Evidence supporting selective dorsal rhizotomy for treatment of spastic cerebral palsy. Cureus.

[33] Park TS, Johnston JM (2006). Surgical techniques of selective dorsal rhizotomy for spastic cerebral palsy. Neurosurg Focus.

[34] Peacock WJ, Staudt LA (1990). Spasticity in cerebral palsy and the selective posterior rhizotomy procedure. J Child Neurol.

[35] Phillips LH, Park TS (1993). The frequency of intradural conjoined lumbosacral dorsal nerve roots found during selective dorsal rhizotomy. Neurosurgery.

[36] Phillips LH, Park TS (1991). Electrophysiologic mapping of the segmental anatomy of the muscles of the lower extremity. Muscle Nerve.

[37] Phillips LH, Park TS, Park TS, Phillips LH, Peacock WJ (1989). Electrophysiologic studies of selective posterior rhizotomy patients. Management of Spasticity in cerebral palsy and spinal cord injury.

[38] Riegle EV, Gunter JB, Lagueruela RG, Park TS, Owen J (1992). Anesthesia for selective dorsal rhizotomy in children. J Neurosurg Anesthesiol.

[39] Rivera ADC, Burke T, Schiff SJ, Weiss IP (1994). An experimental study of reflex variability in selective dorsal rhizotomy. J Neurosurg.

[40] Russell DJ, Rosenbaum PL, Avery LM, Lane M (2003). Gross motor function measure (GMFM-66 and GMFM-88) User’s Manual Clinics in Developmental Medicine No. 159.

[41] Sala F, Squintani G, Tramontano V, Arcaro C, Faccioli F, Mazza C (2013). Intraoperative neurophysiology in tethered cord surgery: techniques and results. Childs Nerv Syst.

[42] Salame K, Ouaknine GE, Rochkind S, Constantini S, Razon N (2003). Surgical treatment of spasticity by selective posterior rhizotomy: 30 years experience. Isr Med Assoc J.

[43] Schirmer CM, Shils JL, Arle JE, Cosgrove GR, Dempsey PK, Tarlov E, Kim S, Martin CJ, Feltz C, Moul M, Magge S (2011). Heuristic map of myotomal innervation in humans using direct intraoperative nerve root stimulation. J Neurosurg Spine.

[44] Shimamura M (1973). Spino-bulbo-spinal and propriospinal reflexes in various vertebrates. Brain Res.

[45] Staudt LA, Nuwer MR, Peacock WJ (1995). Intraoperative monitoring during selective posterior rhizotomy: technique and patient outcome. Electroencephalogr Clin Neurophysiol.

[46] Steinbok P, Tidemann AJ, Miller S, Mortenson P, Bowen-Roberts T (2009). Electrophysiologically guided versus non-electrophysiologically guided selective dorsal rhizotomy for spastic cerebral palsy: a comparison of outcome. Childs Nerv Syst.

[47] Steinbok P, Kestle JR (1996). Variation between centers in electrophysiologic techniques used in lumbosacral selective dorsal rhizotomy for spastic cerebral palsy. Pediatr Neurosurg.

[48] Tan AM, Chakrabarty S, Kimura H, Martin FH (2012). Selective corticospinal tract injury in the rat induces primary afferent fiber sprouting in the spinal cord and hyperreflexia. J Neurosci.

[49] Turner RP (2009). Neurophysiologic intraoperative monitoring during selective dorsal rhizotomy. J Clin Neurophysiol.

[50] Williams P, Jiang YQ, Martin JH (2017). Motor system plasticity after unilateral injury in the developing brain. Dev Med Child Neurol.

[51] Wolter S, Spies C, Murphy J, Michael T, Haberl H (2014). UMN lesion dependent asymmetry?. Clin Neurophysiol.

